# Impact of Supplementation and Nutritional Interventions on Pathogenic Processes of Mood Disorders: A Review of the Evidence

**DOI:** 10.3390/nu13030767

**Published:** 2021-02-26

**Authors:** Cara T. Hoepner, Roger S. McIntyre, George I. Papakostas

**Affiliations:** 1 Bay Area Psychiatric, A Nursing Corporation, San Francisco, CA 94111, USA; 2 Mood Disorders Psychopharmacology Unit, University of Toronto, Toronto, ON M5T 2S8, Canada; Roger.McIntyre@uhn.ca; 3 Massachusetts General Hospital, Boston, MA 02114, USA; gpapakostas@partners.org

**Keywords:** depression, adjunctive therapy, antidepressants, nutritional deficiencies, inflammation, L-acetylcarnitine

## Abstract

This narrative review was conducted using searches of the PubMed/Medline and Google Scholar databases from inception to November 2019. Clinical trials and relevant articles were identified by cross-referencing major depressive disorder (and/or variants) with the following terms: folate, homocysteine, S-adenosylmethionine (SAMe), L-acetylcarnitine, alpha-lipoic acid, N-acetylcysteine, L-tryptophan, zinc, magnesium, vitamin D, omega-3 fatty acids, coenzyme Q10, and inositol. Manual reviews of references were also performed using article reference lists. Abnormal levels of folate, homocysteine, and SAMe have been shown to be associated with a higher risk of depression. Numerous studies have demonstrated antidepressant activity with L-methylfolate and SAMe supplementation in individuals with depression. Additionally, the amino acids L-acetylcarnitine, alpha-lipoic acid, N-acetylcysteine, and L-tryptophan have been implicated in the development of depression and shown to exert antidepressant effects. Other agents with evidence for improving depressive symptoms include zinc, magnesium, omega-3 fatty acids, and coenzyme Q10. Potential biases and differences in study designs within and amongst the studies and reviews selected may confound results. Augmentation of antidepressant medications with various supplements targeting nutritional and physiological factors can potentiate antidepressant effects. Medical foods, particularly L-methylfolate, and other supplements may play a role in managing depression in patients with inadequate response to antidepressant therapies.

## 1. Introduction

Major depressive disorder (MDD) is among the most common and debilitating medical conditions [1]. Since the 1950s, the monoamine theory influenced the development of mainstay antidepressant treatments, including selective serotonin reuptake inhibitors (SSRIs) and serotonin norepinephrine reuptake inhibitors (SNRIs) [2]. However, more recently, other biochemical and physiological factors have been shown to contribute to the pathophysiology of MDD [3]. As a result, there are a growing number of novel MDD treatment options, including nutritional augmentation strategies, that have become available based on this evolving knowledgebase [4].

This narrative review will discuss how nutritional imbalances, in particular, affect pathogenic processes in MDD and other mood disorders and will review evidence for nutritional interventions with an emphasis on essential and conditionally essential nutrients found in the body, including established medical foods, amino acids, and other supplements.

## 2. Proposed Nutritional and Other Novel Contributors to the Pathogenesis of MDD

MDD is a heterogeneous disorder, and accumulating research has revealed multifactorial processes involved in the symptomatology of MDD, beyond dysfunction of monoamine neurotransmitter regulation [5,6,7]. Along with the biopsychosocial, cognitive, and behavioral components of MDD [8]. nutritional imbalances can impact mood and neurological functions [9,10,11,12]. Nutrients, including vitamins, minerals, fatty acids, and essential amino acids, influence neurological hormonal, neurotransmitter, and signaling pathways that modulate brain functions, including cognition and mood [13]. Similar pathways in the gut influence neurotransmitter function and neuroinflammation, which ultimately affect mood [14].

The inflammatory system has been implicated in the onset, phenomenology, and comorbidity of mood disorders [15]. In particular, inflammation can alter mood, energy, sleep, cognition, and motivation, which are key aspects of MDD. Neuroinflammation has been extensively studied in mood disorders and has been implicated in depressive symptoms and neurodegeneration, two commonly comorbid conditions [16]. Inflammation induces neurodegenerative processes, as evidenced by neuronal and glial cell atrophy/loss, in addition to reducing neuroprotection and neuronal repair. It has been suggested that a mechanistic link between inflammation and depression is the impact of cytokines on serotonin levels, glutamate metabolism, the dopamine pathway, the hypothalamic-pituitary-adrenal (HPA) axis, microglial activation, and brain structure. [15,17,18] Increased levels of C-reactive protein ([CRP]; ≥1.0 mg/L), interleukin-6, interleukin-1, and tumor necrosis factor-α (TNF-α) have been repeatedly shown in clinical trials and meta-analyses to have a positive correlation with MDD [15,17,19,20,21,22]. Furthermore, cytokines may interfere with oligodendrocytes, which modulate glutamate transmission, by contributing to glutamate excitotoxicity and axonal damage in the white matter of the brain [23]. This cascade of events ultimately impacts neural plasticity through excitotoxicity, decreased neurogenesis, increased glutamatergic activation, oxidative stress, and induction of apoptosis [18]. These inflammation-mediated changes may have implications for the long-term course of MDD, including response to treatment, so managing inflammation is likely an important aspect of treating MDD. In particular, levels of IL-6 and CRP have been associated with an increased likelihood of experiencing treatment-resistant depression, suggesting that there is a role for anti-inflammatory interventions to be used as adjunctive treatments for patients with MDD [24].

More recently, epigenetic mechanisms have been identified in the pathophysiology of depressive symptoms and potentially increase the risk for developing MDD [24,25]. Epigenetic factors are inherited and acquired mechanisms that regulate gene function, by altering DNA methylation and chromatin structure, without modifying DNA sequence [24]. Nucleosomes, the basic units of chromatin, are formed by wrapping DNA around histone octamers, which can be modified via acetylation to increase gene expression or methylation to activate or repress gene transcription, depending on the amino acid involved [26]. These epigenetic modifications to DNA and histones often occur secondary to stress and may result in downstream effects that exaggerate or reduce depression-like behavior [26,27]. When considering management approaches to target the different mechanisms behind MDD, there is an opportunity to incorporate established medical foods and supplements, and also explore the use of additional products in certain subsets of patients.

## 3. Dysregulation of the One-Carbon Cycle in MDD

### 3.1. Vitamin B_12_ and Folate

Vitamin B_12_ and folate are critical to central nervous system (CNS) development and function by acting as cofactors in converting homocysteine to methionine, an essential amino acid involved in numerous methylation processes critical for synthesizing proteins, lipids, nucleic acids, neurotransmitters, and hormones [28]. Vitamin B_12_ deficiency is known to disrupt infant brain development and cause neural tube defects, supporting its connection to brain function [29]. Altered vitamin B_12_ levels also are associated with issues well beyond infancy, particularly with inflammatory conditions that result in psychiatric disorders, including depressive disorders [10,30,31]. Inflammation and depressive symptoms have a bidirectional relationship, facilitating and promoting one another, as evidenced by elevation in various inflammatory biomarkers in a subgroup of susceptible MDD patients. [19,22,32] From a mechanistic standpoint, inflammation can trigger activation of microglia and subsequently release proinflammatory cytokines, which induce depressive symptoms by altering production, metabolism, and transport of neurotransmitters that affect mood (e.g., dopamine, glutamate, serotonin) [33,34].

**Figure 1:**
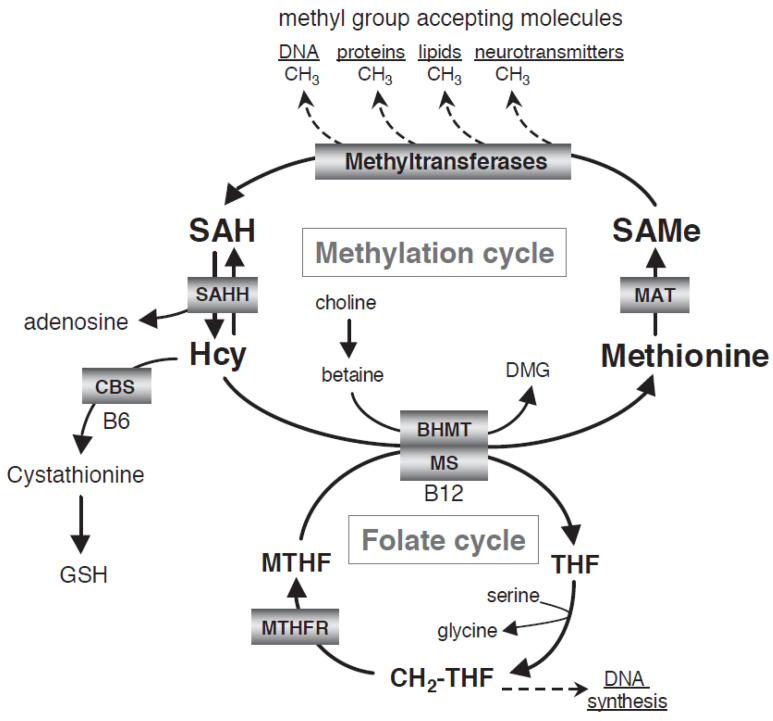
One-carbon folate methylation cycle [38]. BHMT, betaine:homocysteine methyltransferase; CBS, cystathionine-β-synthetase; DMG, dimethylglycine; GSH, glutathione; Hcy, homocysteine; MAT, l-methionine *S*-adenosyltransferase; MTHF, 5-methyltetrahydrofolate; *MTHFR*, 5-methyltetrahydrofolate reductase; SAH, *S*-adenosylhomocysteine; SAHH, *S*-adenosylhomocysteine hydrolase; SAMe, *S*-adenosylmethionine; THF, tetrahydrofolate.

Folate deficiencies may cause depressive symptoms by elevating homocysteine and intracellular one-carbon metabolism (Figure 1) [10,28]. The one-carbon cycle is critical to the numerous transmethylation processes occurring in the CNS and is involved in metabolizing monoamine neurotransmitters such as serotonin, norepinephrine, and dopamine [35,36]. Folic acid must first be converted by 5,10-methylenetetrahydrofolate reductase (MTHFR) to the metabolically active form, L-5-methyl-tetrahydrofolate (MTHF), which is the only form of folate that crosses the blood-brain barrier (BBB). [28,35,37] Upon entering the CNS, MTHF acts as the methyl donor in numerous methylation-dependent processes, including the methylation of homocysteine to form methionine and *S*-adenosylmethionine (SAMe). Methionine is an essential amino acid and acts as the substrate for SAMe, a methyl group donor in more than 100 methylation reactions in the body [28]. Methionine also is a precursor to glutathione, a naturally occurring antioxidant that modulates glutamate activity and maintains cellular oxidative balance by scavenging and neutralizing reactive oxygen and nitrogen species [38,39,40].

Augmenting antidepressants with folate may benefit MDD patients who are predisposed to or already have low folate levels. Folic acid supplementation can improve depressive symptoms, cognition, and oxidative imbalances, and induce hippocampal neurochemical changes [41]. Despite these positive outcomes, L-methylfolate (i.e., L-5-methyl-tetrahydrofolate) may be a better alternative than folic acid due to its ability to pass the BBB [28,37]. Unlike folic acid, L-methylfolate does not carry the risk of masking symptoms of vitamin B_12_ deficiency and may have fewer drugs interactions that inhibit dihydrofolate reductase [37].

Two multicenter, sequential, parallel-comparison trials evaluated the effect of augmentation with L-methylfolate (DEPLIN^®^_,_ Alfasigma USA, Inc., Covington, LA, USA) in patients with SSRI-resistant MDD (Table 1) [42,43]. In Trial 1, there were no significant differences in treatment outcome between the groups. Trial 2 used the same design, but assessed a higher dose of L-methylfolate (15 mg/day; *n* = 75). Patients who augmented their SSRI therapy with L-methylfolate demonstrated a significantly better treatment response with minimal adverse events. Furthermore, post-hoc analyses revealed that patients with certain biomarkers (i.e., high levels of TNF-α, CRP, IL-8, BMI ≥ 30 kg/m^2^) had significant improvements in depressive symptoms with L-methylfolate augmented SSRI treatment [44,45].

MTHFR polymorphisms are also implicated in the development of depressive symptoms and reduce the ability to adequately synthesize monoaminergic neurotransmitters; thus the effectiveness of SSRIs and SNRIs may be limited in these patients [62,63,64]. Real-world evidence of adjunctive L-methylfolate has demonstrated increased response and symptom improvement in patients with treatment-resistant depression [65], particularly in those with the *MTHFR* gene mutation [66], with a favorable safety profile [67]. However, it is important for clinicians to exercise caution when using *MTHFR* gene testing to guide their prescribing strategy, but to rather use it in a confirmatory fashion, as the gene tests may not always be accurate in all cases and may lead to over-methylation with folate supplementation. An American Psychiatric Association research council task force published the following statement in the *American Journal of Psychiatry* in 2018: “They find that, at present, there are insufficient data to support the widespread use of combinatorial pharmacogenetic testing in clinical practice, although there are clinical situations in which the technology may be informative, particularly in predicting side effects [68].”

L-methylfolate is generally safe and well tolerated, and lacks the side effects of other standard-of-care therapies for depression [46]. As an adjunct, L-methylfolate has been shown to cause minimal adverse events [42,46]. Potential candidates for L-methylfolate augmentation include treatment-resistant patients with signs of inflammation, hyperhomocysteinemia, and genetic polymorphisms, or low serum folate values.

### 3.2. Homocysteine

Although homocysteine is not considered a nutritional factor, it is an inflammatory marker that may indicate vitamin B_12_ and folate methylation disturbances [69]. Homocysteine is an amino acid that cannot be obtained through diet and can only be formed and removed through the methylation cycle (Figure 1) [38]. In the methylation cycle, *S*-adenosylhomocysteine (SAH) is converted to homocysteine by SAH-hydrolase within the cell. This conversion is reversible; however, under normal physiologic conditions, homocysteine is quickly removed and concentrations are usually low. One way that low intracellular homocysteine concentrations are maintained is through methylation of homocysteine via receipt of a methyl group from 5-MTHF, which then forms methionine and tetrahydrofolate. This requires vitamin B_12_ for transferring the methyl group. Notably, methylation of homocysteine in the CNS can only occur with MTHF as the methyl donor.

Homocysteine is an indicator of select B vitamin deficiencies, as folate and vitamin B_12_ are involved in the conversion of homocysteine to methionine [38,70]. Hyperhomocysteinemia (i.e., an increase in serum homocysteine concentration above the normal plasma or serum homocysteine level) confers risk for vascular disease, impaired bone remodeling, cancer, Parkinson’s disease, Alzheimer’s dementia, mental retardation, and signs and symptoms of neurological dysfunction, including MDD and schizophrenia [38,70,71,72,73]. Normal blood homocysteine levels are 4–15 µmol/L, while levels above 15 are considered high and levels below 12 are considered low. Optimal homocysteine levels are below 10–12 [74]. Hyperhomocysteinemia will ultimately result in neurotoxicity secondary to impaired methylation, excitotoxicity, oxidative stress, and CNS ischemia [38]. Homocysteine is critical for producing neurotransmitters; therefore, altered homocysteine levels may impact mood [70]. Additionally, hyperhomocysteinemia can increase the permeability of the BBB, and can contribute to numerous cerebrovascular pathologies [75].

Elevated mean (SD) plasma homocysteine levels (17.7 [5.4] μmol/L) have been correlated with higher Hamilton Depression Rating Scale (HAM-D) scores, indicating a link between homocysteine dysregulation and depressive symptoms [10]. These patients also had significantly lower red cell and serum folate levels. Plasma homocysteine levels ≥15.0 μmol/L have been linked with depressive symptoms [76,77,78,79]. Testing homocysteine levels in depressed individuals can identify those with folate or vitamin B_12_ deficiency and determine whether patients would benefit from folate supplementation. Expert recommendations include testing serum levels of vitamin B_12_, folate, and methylmalonic acid (MMA) [80]. Folate and/or vitamin B_12_ supplementation is expected to decrease homocysteine, which may contribute to improved methylation and neurotransmitter metabolism and release [38].

### 3.3. S-adenosylmethionine (SAMe)

SAMe can be considered an initial MDD treatment for patients who prefer complementary or alternative approaches; however, it can induce mania in depressed patients on the bipolar spectrum [28,81,82,83,84]. SAMe is a naturally occurring methyl donor involved in over 100 methyltransferase reactions for critical metabolic pathways, including methylation of DNA bases, proteins, phospholipids, free amino acids, catecholamines, and neurotransmitters. [81,82,84] DNA methylation acts to turn off gene transcription, while demethylation is linked to transcriptional activation; therefore, aberrant methylation can negatively impact CNS disorders [81,85]. SAMe is generated from L-methionine in the one-carbon cycle, which is dependent upon sufficient levels of folate and vitamin B_12_, both of which are linked to depressive symptoms [83].

There are several mechanisms potentially responsible for the antidepressant activity of SAMe. As a donor of methyl groups, SAMe may exert antidepressant effects through the methylation of plasma phospholipids, which results in alteration of the neuronal membrane fluidity and function of proteins in the membrane, including monoamine receptors and transporters [84]. Lower SAMe levels may cause a decrease in monoamine synthesis, thereby increasing risk of depression. In animal models, increased SAMe levels were positively correlated with monoamine neurotransmitter concentrations in the brain [28]. SAMe may be taken orally up to 1600 mg/d, a significantly bioavailable and non-toxic dose [49]. Patients receiving SAMe 800 mg/day in addition to their normal antidepressant have shown significantly greater changes in HAM-D scores from baseline and higher remission rates compared with placebo (*p* < 0.05) (Table 1) [47]. In a systematic review, all studies demonstrated a significantly positive effect for SAMe up to 1600 mg on the HAM-D in MDD [48]. SAMe produces a rapid onset of action, with nearly a 5-point difference in HAM-D scores from baseline to week 1 [47]. In a Cochrane systematic review, 8 trials that included 934 participants investigated the effects of SAMe versus placebo or SSRIs in MDD [49]. Compared with placebo, SAMe was more efficacious as an adjunctive treatment in terms of response and remission; however, the level of evidence was low.

SAMe is generally well tolerated and has a favorable safety profile. Frequently reported adverse events associated with SAMe are nausea, diarrhea, and abdominal discomfort [81]. As previously mentioned, SAMe may induce mania and hypomania, even in patients without a previous history of bipolar disorder [49].

## 4. Role of Amino Acids in MDD

### 4.1. L-acetylcarnitine (LAC)

L-carnitine is an essential nutrient found in almost all tissues of the human body, including the brain [86]. Intracellular carnitine levels are depleted under specific circumstances such as diabetes, hemodialysis, and carnitine deficiency secondary to genetic conditions that requires supplementation [86,87]. L-carnitine facilitates the transfer of activated long chain fatty acids (LCFA) through the carnitine shuttle, which is a series of reactions that transport fatty acids into the mitochondria, as acyl-carnitine ester, for β-oxidation. The carnitine shuttle prevents buildup of harmful LCFA and long chain acyl-coenzyme A (acyl-CoA) [86]. Carnitine also assists with transferring toxic compounds out of the mitochondria [87].

LAC is the acetyl derivative of L-carnitine with 2 carbons in the acyl moiety and is commonly found in plasma and body tissue [87]. Carnitine, along with LAC, passes through the BBB and accumulates in the cerebral cortex, of which 10–15% is the LAC moiety. LAC is important for metabolic processes, such as modulating glucose metabolism, stimulating glycogen synthesis, increasing plasma adenosine triphosphate (ATP) concentration, and improving neurological function.

Although the exact mechanism of LAC for managing depressive symptoms is unclear, it has been hypothesized that its neuroplasticity effect, neurotransmitter regulation, and metabotropic glutamate (mGlu) receptor upregulation likely contribute [86,88]. LAC has neuroprotective, anti-inflammatory, and antioxidant properties that also may improve depressive symptoms. In animal depression models, LAC rapidly improved depressive-like behaviors, restored glutamate levels, and increased type 2 mGlu (mGlu2) expression through epigenetic modification, specifically histone acetylation [25]. In a recent study, lower LAC levels were reported in MDD patients compared with controls (Table 1) [50]. Furthermore, lower LAC levels in patients with MDD were associated with greater severity and earlier onset of depressive symptoms. A significantly greater proportion of patients with treatment-resistant depression had a decrease in LAC levels (*p* = 0.01), indicate a potential for augmenting antidepressants with LAC. A meta-analysis of studies investigating the effects of LAC on depressive symptoms showed that LAC was significantly decreased depressive symptoms compared with placebo (standardized mean difference [SMD] −1.10; 95% confidence interval [CI] −1.65, −0.56; *p* < 0.001) [51]. Furthermore, patients taking LAC had a comparable rate of adverse events (AEs) as those taking placebo/no interventions (odds ratio [OR] 0.86; 95% CI: 0.46, 1.63; *p* = 0.648) and a significantly lower risk of AEs compared with antidepressants (OR: 0.21; 95% CI: 0.12, 0.36; *p* < 0.001), with lower rates of gastrointestinal and nervous system AEs [51]. Further investigation is needed to fully characterize the safety of LAC due to the limited data [87]. Individuals who may benefit from LAC supplementation are likely those with lower LAC levels and elevated inflammatory markers.

### 4.2. Alpha-Lipoic Acid (ALA)

ALA is a lipoamide that is synthesized from octanoic acid in the mitochondria and can be obtained through the diet [89]. Once absorbed from the diet, ALA is reduced to dihydrolipoic acid (DHLA) and accumulates mainly in skeletal muscle, liver, and heart, and can cross the BBB [89,90]. ALA plays an important role in mitochondrial energy metabolism by acting as a necessary cofactor for mitochondrial α-ketoacid dehydrogenase reactions [89,91,92]. Additionally, ALA functions as an antioxidant by scavenging reactive oxidative species; chelating transition metals (e.g., iron, copper); and enhancing activity and synthesizing endogenous antioxidants or antioxidant enzymes. DHLA is among the most potent naturally occurring antioxidants and can regenerate other endogenous antioxidants, neutralize free radicals, and chelate metals that contribute to oxidative stress [89]. ALA also significantly reduces cytokine-induced inflammation by decreasing production of IL-6, IL-1β, and TNF-α (*p* < 0.05) [93,94]. Individuals receiving ALA 300 mg experienced a 15% significant reduction in IL-6 levels compared with placebo (*p* < 0.001) [89,95].

ALA has been investigated as augmentation therapy for MDD, given its biological properties and potential role in the pathophysiological factors involved in mood disorders (Table 1). Augmenting desvenlafaxine with ALA in mice was associated with significantly greater improvement in depressive symptoms compared with either treatment alone, demonstrating a potentiating effect of these products together [96]. Combination treatment of ALA and LAC also has been studied in MDD in light of their role in modulating mitochondrial function and metabolism, and neuroprotective effects; however, studies have reported inconsistent results [87]. Together, LAC and ALA have been shown to reduce the number of damaged neuronal mitochondria and increase intact hippocampal mitochondria, thereby improving brain function [97,98]. In preclinical studies, LAC and ALA reversed age-related increase in oxidants, reduced oxidative damage in the brain, and improved metabolic rate and physiological activity without causing further oxidation [99]. Conversely, a randomized controlled trial of LAC plus ALA versus placebo in humans reported no significant differences in Montgomery–Åsberg Depression Rating Scale (MADRS) score between groups [52].

### 4.3. N-acetylcysteine (NAC)

NAC, the acetyl derivative of cysteine, is a glutathione precursor that is known as an antidote for paracetamol overdose [100,101]. A critical role of NAC is restoring cellular glutathione concentrations by providing cysteine in glutathione production [39]. The brain is susceptible to various reactive oxygen species that can cause oxidative cellular dysfunction; thus, oxidative stress is implicated in the pathogenesis of mood disorders [101]. In addition to glutathione replenishment, NAC has been shown to have anti-inflammatory activity by reducing inflammatory cytokines in the brain, which is a potential mechanism for how NAC exerts antidepressant effects [39,102]. NAC plays a role in neurotransmission by modulating glutamate pathways and regulating dopamine release [39].

Recently, NAC has emerged as a potential supplemental treatment for psychiatric and neurological disorders, including MDD (Table 1). A significantly greater proportion of MDD patients receiving NAC responded to treatment and reached remission (*p* < 0.05) in a study comparing adjunctive NAC with placebo [55]. In a meta-analysis conducted by Fernandes et al., NAC improved depressive symptoms and functionality compared with placebo over a follow-up of 12–24 weeks; however, NAC did not improve quality of life [54]. In another meta-analysis of adjunctive NAC in MDD, there was no significant difference in efficacy between NAC and placebo, but individuals treated with adjunctive NAC showed a positive trend towards efficacy, especially in those with higher MADRS scores [53]. Notably, patients in the NAC group reported more gastrointestinal (33.9% vs. 18.4%; *p* = 0.005) and musculoskeletal complaints compared with placebo (3.9% vs. 0%; *p* = 0.025). Furthermore, adjunctive NAC has been demonstrated to improve symptom severity, function, and quality of life in MDD and major depressive episodes in bipolar disorder [55,56]. Doses ranging from 1–3 g have been studied in patients with MDD [54,55,101,103] Doses of 2400–3000 mg/day have been found to be safe and effective for obsessive-compulsive and related disorders [103], NAC is generally well tolerated with no severe AEs reported in studies [101]. The most common AEs associated with NAC were gastrointestinal, neurological, psychological, musculoskeletal, and dermatological. Individuals with glutathione deficiency may be potential candidates to supplement their current antidepressant treatment with NAC.

### 4.4. L-tryptophan

L-tryptophan is an essential amino acid used for protein synthesis and serotonin biosynthesis [104]. Tryptophan undergoes degradation through the kynurenine pathway, where it is converted to kynurenine and ultimately serotonin. It has been suggested that an impairment of neuroprotective components of the kynurenine metabolic pathway plays a role in depression, as evidenced by lower tryptophan availability, higher tryptophan breakdown, and lower mean plasma kynurenic acid concentration in MDD patients [105]. Alterations in tryptophan levels can impact serotonin synthesis and mood [106]. An increase in HAM-D score was reported following acute tryptophan depletion in patients with a history of MDD [107]. Another study also reported significantly increased HAM-D scores (*p* < 0.0009) in medication-free remitted MDD patients [108]. Low tryptophan levels and increased levels of its detrimental catabolites, kynurenine and quinolinic acid, in plasma are associated with the development of depressive disorders [104,109]. Additionally, elevations in IL-6 in models of depressive symptoms are due to increased HPA activity, thereby cortisol and activating tryptophan 2,3-dioxygenase (TDO), which produces more tryptophan catabolites and less serotonin [110]. In the presence of inflammation, tryptophan produces kynurenic and quinolinic acid; therefore, it must be used with caution in patients with inflammation.

High L-tryptophan doses can result in mild nausea, tremor, dry mouth, and dizziness [111,112]. Tryptophan should be used cautiously with monoamine oxidase inhibitors and SSRIs, which increase the risk of serotonin syndrome, and occurs when there is excessive synaptic serotonin in the brain [112,113]. Serotonin syndrome typically presents with tremor, hyperreflexia, autonomic irregularities, and change in mental status (e.g., agitation, restlessness, delirium, confusion) [113]. Other serotonin-elevating drugs include SSRIs, SNRIs, tricyclic antidepressants, St. John’s wort, and pain medications.

Despite the compelling evidence from tryptophan depletion studies that suggest that tryptophan is associated with depressive symptoms, [104,107,109] the actual relationship between tryptophan and the pathophysiology of MDD has not been established. Furthermore, tryptophan must be used with caution when taken with medications that increase serotonin, a common mechanism of action of numerous standard-of-care treatments for MDD. In addition to being mindful of concomitant medications, prescribers and patients must consider the 2- to 3-times daily dosing that may pose a compliance issue.

## 5. Minerals

### 5.1. Zinc

Zinc is an essential trace element that is involved with a number of vital CNS biochemical and physiological processes, thereby facilitating proper brain development and function [114,115]. Zinc primarily acts as a cofactor for over 300 enzymatic processes and is involved in gene transcription and replication, DNA repair, cell growth, neurogenesis, neuronal development, maintaining oxidative balance, and protein synthesis [115,116]. Additionally, zinc is a modulator of immune and inflammatory processes, and affects inflammatory cytokine levels [115]. Importantly, zinc has been identified as an antagonist of the N-methyl-D-aspartate (NMDA) receptor, thereby downregulating glutamate response. [114,116,117] Maintaining appropriate zinc levels is critical in brain regions involved in depressive symptoms including the cerebral cortex, hippocampus, and amygdala [114]. Depleted zinc levels enhance HPA activity, which causes a surge in glucocorticoids, ultimately inducing hippocampal dysfunction and behavioral abnormalities [118]. Hyperactivation of the HPA axis can cause an imbalance in serotonergic and noradrenergic circuits, affecting mood [33,118]. Zinc deficiency may contribute to developing depressive disorders by increasing cortisol levels, decreasing neurogenesis and neuroplasticity, and disturbing glutamate homeostasis [114].

Zinc deficiency has been shown to be significantly associated with MDD and depression symptom severity [59,115]. In a study evaluating zinc supplementation to imipramine, there was an inverse correlation between zinc concentrations and MADRS score. A greater proportion of patients achieving remission had significantly higher zinc concentrations after 12 weeks of supplementation [57]. Higher zinc intake in patients receiving SSRI resulted in nearly a 50% decrease in depressive symptoms (*p* = 0.007) compared with those with lower zinc intake [58]. Studies from a meta-analysis also reported an association of greater mean symptom severity with greater differences in zinc between patients with MDD and controls [59]. Furthermore, patients with treatment-resistant depression were found to have lower zinc concentrations than treatment-non-resistant patients. [57,115,117] Given these findings, supplemental zinc may be a beneficial adjunct to antidepressants, and obtaining zinc blood concentrations may potentially be a biological marker for monitoring MDD severity.

### 5.2. Magnesium

Magnesium is an essential mineral that functions as a cofactor for >600 enzymes involved in a variety of physiological processes, including the production of vitamin D [119,120]. Magnesium levels can impact CNS function, as it plays a role in DNA replication, transcription, and translation [114]. Magnesium also is recognized for its ability to antagonize the NMDA glutamate receptor, the mechanism thought to be behind its antidepressant and neuroprotective effects [114,121]. Magnesium deficiency causes NMDA hyperactivity and consequently leads to the development of depressive and anxiety-like symptoms and increased inflammatory markers [122]. Low magnesium intake has been linked to an increased risk of experiencing depressive symptoms (OR 1.73, 95% CI 1.48, 2.02; relative risk [RR] 1.49, 95% CI 1.35, 1.66) [123]. Antidepressant action of magnesium has been reported in both animal and human studies [60,124,125,126]. Consuming magnesium significantly improved depression and anxiety scores (Table 1) [60]. In addition, 61% of patients reported a positive experience with magnesium and would continue taking it for mood.

## 6. Other Supplementation

### 6.1. Vitamin D

Vitamin D plays a critical role in a number of physiologic processes, such as muscle function, regulating cell growth, cancer prevention, metabolic signaling, inflammation, and autoimmunity [127,128]. Vitamin D is integral in a number of brain processes including neuroimmunomodulation, neuroplasticity, neuroprotection, and brain development, which suggests its potential link to depressive disorders [129]. It is thought that vitamin D may affect brain function by acting on vitamin D receptors (VDRs) located in the CNS [130]. VDRs are found in various regions of the brain involved in depressive symptoms including the hypothalamus, prefrontal cortex, hippocampus, thalamus, and substantia nigra. *VDR* gene polymorphisms have been shown to be associated with cognitive and behavioral impairment, and increased anxiety [128,130]. Potential actions of vitamin D in the brain include neurotrophin stimulation, antioxidation, and anti-inflammation by inhibiting the release of cytokines and metalloproteinases [128]. Additionally, vitamin D promotes glutathione metabolism in neurons, providing protection from oxidative degeneration [127].

Vitamin D deficiency has been shown to be associated with the presence of mood disorders and reduced cognitive functioning. [127,129,131] In a cross-sectional study, patients with serum 25-hydroxyvitamin D levels <10 ng/mL had a significantly greater likelihood of developing a mood disorder (OR: 11.7; 95% CI 2.04, 66.9) than those with adequate vitamin D levels (Table 1) [131]. Additional support is provided by a meta-analysis that demonstrated that low vitamin D levels are associated with a significantly increased risk for depressive symptoms (HR 2.2; 95% CI 1.4, 3.5; *p* < 0.001) [129]. Patients with vitamin D deficiencies not only have a higher risk of depression, but also have greater duration and severity of depressive symptoms [132].

Numerous studies have demonstrated the effectiveness of vitamin D supplementation in improving depressive symptoms [61]. In a meta-analysis of randomized controlled trials evaluating the effect of vitamin D supplementation (≥800 IU daily) on depressive symptoms, 10 of 15 studies reported significant improvement. When the studies were analyzed to exclude those with biological flaws, there was a significant effect size (SMD: 0.78).

Vitamin D toxicity rarely occurs but is caused by consuming excessively high doses. Doses >50,000–100,000 IU/day can cause hypercalcemia and hyperphosphatemia [133,134]. Currently, the tolerable upper intake level of vitamin D is 2000 IU/day; however, doses up to 4000 IU/day have been shown to carry low risk of hypercalcemia [134,135]. Vitamin D toxicity typically manifests as nausea, dehydration, and lethargy [135]. Doses of vitamin D 400–18,400 IU/day have been studied in depression [61].

Although the exact mechanism behind vitamin D deficiency in depressive disorders is not clear, supplementing vitamin D carries low risk, given the overall health benefits of vitamin D and low toxicity at doses of 1000–2000 IU. Experts recommend using 10,000 IU, then retesting and adjusting in order to reach adequate vitamin D levels.

### 6.2. Omega-3 Fatty Acids

Omega-3 fatty acids are recognized for their multitude of health benefits due to their anti-inflammatory, antiarrhythmic, antithrombotic, and hypolipidemic effects [136]. Omega-3 polyunsaturated fatty acids (PUFAs) can only be obtained through the diet and are synthesized by consuming short-chained omega-3 fatty acids, which produce eicosapentaenoic acid (EPA) and docosahexaenoic acid (DHA) [137]. EPA and DHA are involved with different processes related to brain function [136]. DHA is responsible for maintaining the structural integrity of the phospholipid in neuronal cell membranes. Low levels of DHA cause abnormalities in the brain that impact neuron size, nerve growth factor levels, auditory and olfactory responses, and learning and memory [138]. EPA has important physiological functions such as modulating cytokines that affect neurotransmission and neuromodulation [136]. EPA also is thought to reduce inflammation by decreasing IL-1 and TNF-α levels, and also inhibits the upstream mitogen activated protein kinase (MAPK) pathway [137].

Omega-3 PUFAs are thought to improve depression through its role in the uptake, release, metabolism, and receptor function of serotonergic and dopaminergic transmission [139]. Additionally, the anti-inflammatory actions of omega-3 PUFAs are an important mechanism that may address depression-related inflammation. Omega-3 PUFAs, including DHA and EPA, have been found to modulate and reduce neuroinflammation [140,141]. In a rodent study, omega-3 PUFA deficiency was correlated with increases in the proinflammatory cytokines IL-6 and TNF-α, and CRP [142]. In humans, omega-3 PUFAs also were found to be lower in patients with depressive symptoms compared with non-depressed individuals [143,144]. In a study conducted by Rapaport et al., MDD patients who had high levels of inflammation (as measured by hs-CRP, IL-1RA and IL-6), experienced greater improvement with EPA than placebo or DHA, while those who received DHA experienced less improvement than placebo [145]. Patients without any high inflammatory biomarkers experienced a decreased response to EPA than those receiving placebo or DHA. By week 8, patients with high biomarkers who received EPA had at least an 11-point decrease in HAM-D scores, compared to those who received placebo who were progressively less responsive and had increases in inflammatory biomarkers. Despite these positive findings, in a recent meta-analysis, supplementation with omega-3 PUFAs showed little to no effect on risk of depression or anxiety symptoms (number needed to harm, 1000), and inconclusive findings on its effects on depression symptom severity and risk of remission [146]. Patients with increased inflammation and comorbid inflammatory diseases may benefit from omega-3 PUFAs; however, there is conflicting evidence for its use in depressive disorders.

### 6.3. Coenzyme Q10 (CoQ10)

CoQ10, also known as ubiquinone, is a potent antioxidant that possesses anti-inflammatory and neuroprotective properties [147,148]. It protects cells from reactive oxygen and nitrogen species by regenerating oxidized tocopherol and ascorbate, and also enhances mitochondrial activity in the brain [148]. CoQ10 also is involved in a number of biological roles such as cellular membrane repair, regulation of inflammation, and gene expression. As such, reduced levels of CoQ10 in the body are associated with increased free radicals and free radical damage, along with decreased mitochondrial energy production. Patients with MDD are associated with significantly lower plasma CoQ10 levels than healthy individuals [147]. Furthermore, patients with treatment-resistant depression were found to have significantly lower CoQ10 levels than those who were not treatment resistant. In patients with bipolar disorder, supplementing their psychotropic medications with CoQ10 resulted in a significant decrease in MADRS score from baseline after 4 weeks, with significant changes seen as early as 2 weeks [149]. CoQ10 is generally well tolerated and associated with minimal severe AEs. Clinicians should consider supplementing antidepressant regimens with CoQ10 in patients with treatment-resistant depression.

## 7. Conclusions and Future Directions

Conventional antidepressant treatment options do not adequately meet the needs of all patients with depressive disorders, as they do not directly address underlying pathogenic factors including nutritional deficiencies, inflammation, oxidative stress, neuroprotection, and neurogenesis. Additionally, there are limited treatment options for patients with MDD that are resistant to conventional treatments. Targeting nutritional imbalances and signaling abnormalities using medical foods and dietary supplements provides several augmentative strategies to treating MDD in patients who do not sufficiently respond to antidepressants and mood stabilizers. Current evidence suggests that medical foods, particularly L-methylfolate and LAC, and other supplements have a role in the adjunctive treatment of MDD, and may particularly target aspects of inflammation and other factors contributing to the pathophysiology of depressive symptoms (Table 2). [48,51,53,59,65,129,146,149].

Augmenting conventional antidepressants with medical foods may be a viable option for individuals with MDD who have tried and failed multiple antidepressant regimens and/or are resistant to conventional antidepressants. In particular, L-methylfolate has the most robust body of evidence to support its use in this area. However, the studies that were reviewed have some potential limitations, including small sample sizes, use of various depression ratings scales, and enrollment of heterogeneous populations, so additional research is needed to fully explore potential synergies between agents during the management of MDD. Of particular interest may be the effect of these interventions on those who are treatment resistant or are experiencing residual depressive symptoms with antidepressant monotherapy. Augmentation strategies that manage some of the underlying factors that are not effectively affected by traditional antidepressant treatments may be of particular interest to this population.

## Figures and Tables

**Table 1:** Review of the clinical evidence for supplementation in depression.

Study	Design	Size	Efficacy	Safety
**L-methylfolate**				
Ginsberg et al. 2011. [46]	Retrospective analysis of L-methylfolate as adjunctive therapy to SSRI/SNRI in patients with MDD	242 patients	L-methylfolate in addition to SSRI/SNRI therapy was superior to SSRI/SNRI monotherapy in improving depressive symptoms and functions (CGI severity reduction ≥ 2: 19% vs. 7%; *p* = 0.01) within 60 days	There were no major differences in adverse events between the two groups. The most commonly reported adverse events included sexual dysfunction, somnolence, nausea, dizziness, insomnia, agitation, constipation, and fatigue.
Papakostas et al. 2012. [42]	Two randomized, double-blind, parallel-sequential trials Trial 1: Patients with SSRI-resistant MDD were randomized to placebo or L- methylfolate 7.5 mg/day for 60 days, or placebo for 30 days and then L- methylfolate as adjunctive to SSRIs Trial 2: Patients with SSRI-resistant MDD were randomized to placebo or L- methylfolate 15 mg/day for 60 days, or placebo for 30 days and then L- methylfolate as adjunctive to SSRIs	Trial 1: 148 patients Trial 2: 75 patients	In Trial 1, 7.5 mg adjunctive L-methylfolate was not superior to placebo; however, Trial 2 demonstrated that L-methylfolate 15 mg was associated with a higher response rate than placebo (32% vs. 15%; *p* = 0.05) and significant improvement on the QIDS-SR score and CGI severity scale.	Comparable side effect profile with placebo; most common side effect categories were gastrointestinal (17%), somatic (14%), and infectious (11%)
Papakostas et al. 2014. [44]	Post hoc analysis of Papakostas et al. 2012.	74 patients	Patients with genetic markers at baseline showed a greater mean change from baseline on the 28-item HAM-D (*p* < 0.05) and response rate (*p* < 0.05) with L-methylfolate compared with placebo. Genetic markers with the greatest mean change from baseline were *MTHFR* 677 CT/TT + *MTR* 2756 AG/GG, GCH1TC/TT + *COMT* GG, and *GCH1* TC/TT + *COMT* CC.	N/A
Shelton et al. 2015. [45]	Exploratory, post-hoc analysis of Papakostas et al. 2012.	74 patients	Significant changes in mean change on the 17-item HAM-D were reported with L-methylfolate versus placebo (*p* < 0.05) for those with greater than median baseline levels of TNF-α, IL-8, hsCRP, and leptin. Patients with BMI ≥30 kg/m^2^ with TNF-α, IL-6, IL-8, IL-12, hsCRP, and leptin had statistically significant treatment effects versus placebo (*p* ≤ 0.05).	N/A
***S*-adenosylmethionine (SAMe)**				
Papakostas et al. 2010. [47]	Single-center, randomized, double-blind study of SAMe augmentation of SRIs in nonresponding patients with MDD	73 patients	Four patients discontinued placebo and two discontinued SAMe due to inefficacy. Based on HAM-D scores, 18 patients in the SAMe group responded and 14 remitted, compared with 6 patients in the placebo group who responded and 4 who remitted (*p* = 0.01, and *p* = 0.02, respectively). Based on CGI ratings, remission and response rates were greater in SAMe-treated patients versus placebo-treated patients.	Three placebo and two SAMe patients discontinued treatment due to intolerance of treatment. No statistically significant differences in adverse events were reported, although there was a marginally higher mean supine systolic blood pressure reading in the SAMe arm (mean difference 3.1 mm Hg). No serious adverse events were reported.
Williams et al. 2005. [48]	Systematic review of studies, reviews, case reports, and pilot projects investigating SAMe in depression among humans	11 studies (5 intervention trials, 2 randomized clinical trials, 2 reviews, 1 controlled clinical trial, 1 meta-analysis)	All intervention studies and randomized trials favored oral SAMe to placebo and had significant effect on the HAM-D.	N/A
Galizia et al. 2016. [49]	A Cochrane systematic review conducted to investigate SAMe as monotherapy and adjunctive in the treatment of MDD in adults	8 clinical trials comparing SAMe with placebo, imipramine, desipramine, or escitalopram in 934 adults	Overall, there was low quality evidence. Based on change from baseline in HAM-D and MADRS score, there was no strong evidence of a difference between the SAMe and placebo groups (SMD −0.54, 95% CI −1.54 to 0.46, *p* = 0.29), along with SAMe and escitalopram (MD 0.12, 95% CI −2.75 to 2.99, *p* = 0.93). Low quality evidence suggested comparable change in depressive symptoms between SAMe and imipramine monotherapy (SMD −0.04, 95% CI, −0.34 to 0.27; *p* = 0.82). Additionally, low quality evidence showed that SAMe was superior to placebo as adjunctive treatment to SSRIs (MD −3.90, 95% CI −6.93 to −0.87, *p* = 0.01).	2 incidences of mania/hypomania of 441 participants receiving SAMe
**L-acetylcarnitine (LAC)**				
Nasca et al. 2018. [50]	Translational study of evaluating the role of LAC in MDD	116 participants	Mean concentration of LAC in patients with MDD were significantly lower than that of healthy controls (6.1 μmol/L ± 0.3 vs. 8.3 μmol/L ± 0.4, respectively; *p* < 0.0001). There was an inverse correlation between severity of MDD based on 17-item HAM-D scores and ALCAR concentrations (*p* = 0.04, r = 0.35). LAC also was shown to be predictive of moderate to severe MDD (*p* = 0.04). Furthermore, earlier age of onset of depression correlated with lower concentration of LAC (*p* = 0.04). Additionally, patients with MDD and a history of TRD were associated with a decrease in LAC levels.	N/A
Veronese et al. 2018. [51]	Systematic review and meta-analysis of 12 randomized controlled trials	393 patients who received LAC and 398 controls	Administration of LAC was associated with a significant reduction of depressive symptoms using various outcomes with an emphasis on the HAM-D compared with controls (SMD −1.10; 95% CI −1.65 to −0.56; *p* < 0.001), although there was some evidence of publication bias (Egger test, −6.69 ± 2.65; *p* = 0.040). Higher LAC doses resulted in better test results when assessing depressive symptoms (*p* = 0.01). LAC showed a similar effect on treating depressive symptoms compared with conventional antidepressants (SMD 0.06; 95% CI −0.22 to 0.34; *p* = 0.686)	Patients receiving LAC had a similar frequency of adverse effects compared with those on placebo, but showed a 79% reduction in adverse effects when compared with antidepressants (OR 0.21; 95% CI 0.12–0.36; *p* < 0.001)
**Alpha-Lipoic Acid (ALA)**				
Brennan et al. 2013. [52]	A randomized, placebo-controlled trial of LAC and ALA versus placebo as augmentation treatment in those with inadequate response to standard treatments for bipolar depression	68 participants	There were no significant differences in mean MADRS score found between LAC/ALA and placebo.	Most frequently reported adverse events were diarrhea (30%), foul-smelling urine (25%), rash (20%), constipation (15%), and dyspepsia (15%)
**N-acetylcysteine (NAC)**				
Zheng et al. 2018. [53]	Meta-analysis of randomized controlled trials of NAC vs. placebo in patients with schizophrenia, bipolar disorder, or MDD	Schizophrenia: 3 trials, *n* = 307 Bipolar disorder: 2 trials, *n* = 125 MDD: 1 trial, *n* = 269	In patients with MDD, there were no significant differences in clinical efficacy between add-on NAC and placebo based on the MADRS.	Patients in the NAC group experienced more gastrointestinal (33.9% vs. 18.4%; *p* = 0.005) and musculoskeletal (3.9% vs. 0%; *p* = 0.025) compared with placebo
Fernandes et al. 2016. [54]	A meta-analysis of double-blind, randomized controlled trials of NAC compared to placebo	5 studies; 574 participants	Adjunctive NAC resulted in moderate improvement in MADRS and HAM-D scores (SMD 0.37; 95% CI 0.19–0.55; *p* < 0.001), but consistently better scores on the CGI-S at follow-up compared with placebo (SMD 0.22; 95% CI 0.03–0.41; *p* < 0.001).	Incidences of severe adverse events were similar between placebo and NAC groups (OR 1.04; 95% CI 0.43–2.51; *p* = 0.920). NAC was associated with an increase in minor adverse events (OR 1.61; 95% CI 1.01–2.59; *p* = 0.049). Frequently reported minor adverse events were gastrointestinal issues such as nausea and heartburn, and musculoskeletal issues such as back and joint pain.
Berk et al. 2014. [55]	A double-blind, randomized, placebo-controlled trial comparing adjunctive NAC with placebo in the acute treatment of moderate to severe MDD	252 participants	Over the course of the study, NAC-treated and placebo-treated patients had similar MADRS scores; however, at week 16, there was significantly greater response in the NAC group than placebo (36.6% vs. 25.0%, respectively; *p* = 0.027). There was a higher likelihood of reaching remission with NAC than with placebo (17.9% vs. 6.2%, respectively; *p* = 0.017). Furthermore, a significantly greater proportion of patients in the NAC-treated group had reduction of symptom severity (*p* = 0.001) and greater improvements in functioning (*p* = 0.001) than placebo.	N/A
Magalhaes et al. 2011. [56]	Secondary exploratory analysis of NAC in bipolar depression	17 participants	Compared with placebo, NAC was associated with significant improvements in symptom severity, function, and quality of life. 80% of NAC-treated patients (*n* = 8) had a 50% reduction in MADRS scores compared with 1 patient in the placebo group with the same outcome (OR 24, 95% CI 1.74–330.80, *p* = 0.015).	Side effects were minor and included headache, abdominal pain, and diarrhea
**Zinc**				
Siwek et al. 2010. [57]	Placebo-controlled, double-blind study of adjunctive zinc in patients receiving imipramine for MDD	60 patients	Treatment-resistant patients demonstrated lower concentrations of zinc than treatment-non-resistant patients. Zinc levels were inversely correlated with MADRS score (*p* = 0.001). Patients who reached remission were found to have a significantly higher zinc level compared to those who had not reached remission.	N/A
Maserejian et al. 2012. [58]	An analysis of cross-sectional, observational epidemiological data from a population-based, random stratified cluster sample survey from 2002 through 2005	3708 patients	Among women with low dietary zinc intake, there was an 80% increased risk of having depressive symptoms (CES-D) compared to those with high dietary zinc intake (P_trend_ = 0.004) ^a^ and those taking supplemental zinc had a lower probability of having depressive symptoms (P_trend_ = 0.03). In women, the odds of ongoing depressive symptoms among SSRI users reduced by half (OR 0.44, 95% CI 0.24–0.80, *p* = 0.007) in those with moderate-to-high zinc intake (OR 2.05, 95% CI 1.28–3.28, *p* = 0.003), compared to those with low zinc intake <12.8 mg/day (OR 4.01, 95% CI 2.56–6.29, *p* < 0.0001).	N/A
Swardfager et al. 2013. [59]	A meta-analysis of zinc concentrations in depression	23 studies	Mean peripheral blood zinc concentrations were 1.85 μmol/L lower in depressed patients versus controls (95% CI −2.51 to −1.19, *p* < 0.00001). In studies examining depressive symptom severity using numerous scales, greater mean depressive symptom severity was associated with greater differences in zinc between depressed patients and controls.	N/A
**Magnesium**				
Tarleton et al. 2017. [60]	Randomized, open-label, crossover study evaluating the effects of magnesium supplementation on symptom improvement in mild-to-moderate depression	126 patients	Unadjusted PHQ-9 depression scores improved with magnesium supplementation (−4.3 points, 95% CI −5.0 to −3.6), with a net improvement of −4.2 points. Unadjusted GAD-7 anxiety scores also improved with magnesium (−3.9 points, 95% CI −4.7 to −3.1), with a net improvement in anxiety of −4.5 points.	The most common side effect was diarrhea, which was reported by 8 participants.
**Vitamin D**				
Spedding et al. 2014. [61]	A systematic review of vitamin D supplementation in depression	15 articles	Of the 15 articles, two studies were identified to be without flaws, which showed a statistically significant positive effect of vitamin D in depression of 0.78 (CI 0.24 to 1.27). Among the studies with biological flaws, there was a statistically significant negative effect of vitamin D with an effect size of −1.1 (CI −0.7 to −1.5). Various ratings scales were used in these studies.	

ALA, alpha-lipoic acid; BMI, body mass index; CI, confidence interval; CES-D, Center for Epidemiological Studies—Depression; CGI, Clinical Global Impressions; CGI-S, Clinical Global Impressions-Severity of Illness; *COMT*, catechol-*O*-methyltransferase; GAD-7, Generalized Anxiety Disorders-7; *GCH1*, GTP cyclohydrolase 1; HAM-D, Hamilton Depression Rating Scale; hsCRP, high-sensitivity C-reactive protein; IL, interleukin; LAC, L-acetylcarnitine; MD, mean difference; MDD, major depressive disorder; *MTHFR*, methylenetetrahydrofolate reductase; *MTR*, methionine synthase; NAC, N-acetylcysteine; PHQ-9, Patient Health Questionnaire-9; QIDS-SR, Quick Inventory of Depressive Symptomatology-Self-Rated; SAMe, *S*-adenosylmethionine; SMD, standardized mean difference; SRI, serotonin reuptake inhibitor; SSRI, selective serotonin reuptake inhibitor; TNF-α, tumor necrosis factor α; TRD, treatment-resistant depression. ^a^ Linear tests for trend were assessed using the median values of deciles of intake to represent the exposure of all participants in the same decile.

**Table 2:** Practical considerations for dietary approaches to management of mood disorders.

Supplemental Agent	Considerations and Guidance
L-acetylcarnitine	Through its inhibition of metabotropic Glu receptor mGluR-2, it blocks glutamate release and has a neuroprotective effect
Alpha-lipoic acid	Beneficial in those with insulin resistance Synergistic effects when it is used with L-acetylcarnitine Reduces corticosteroid-induced BDNF alterations
CoQ10	Provides mitochondrial support for bipolar depression, multiple sclerosis, and depression
Folic acid/L-methylfolate	Despite normal levels of folate, some patients may not be able to methylate appropriately Patients with high homocysteine levels and normal vitamin B_12_ and MMA levels will particularly benefit from L-methylfolate augmentation Identification of *MTHFR* variants is confirmatory, not directive, due to the potential impact of epigenetics and mosaicism
Homocysteine	Levels are universally applicable and are not limited to diagnosis of depression Availability in the outpatient setting allows patients to have more access to testing Blood levels alone are not indicative of folate; therefore, vitamin B_12_ and MMA levels also are used In patients with abnormal homocysteine levels and normal vitamin B_12_ and MMA levels, folates with cofactors may be administered and titrated as tolerated. Patients who are unable to tolerate folate due to side effects should receive folinic acid with cofactors. In patients who cannot take folinic acid, TMG (betaine) may be used Reduction in homocysteine levels increase glutathione
Inositol	Useful for its calming effects in general anxiety disorder, though it may be used in depression for those with improved anxiety following its use Numerous metabolic effects that may be used to improve or reduce depressive symptoms
Iron (ferritin, total iron binding capacity)	Indicative of dopaminergic neurotransmission and methionine system function Transferrin levels are measured if there is a history of deficiency
L-tryptophan	As an amino acid, it is given three times daily, 30 min before or 2 h after food and lasts for a duration of 4–6 h Requires coadministration of cofactors Primarily used for irritability and for carbohydrate cravings, with limited effectiveness compared to other interventions L-tryptophan in the presence of inflammation will lead to quinolinic and kynurenic acid production
Magnesium	Necessary component for conversion of cholecalciferol (vitamin D3) to ergocalciferol (vitamin D2) Magnesium is involved in approximately 800 enzymatic systems for structural functions
NAC	May be used for a number of psychological disorders and conditions including OCD, bipolar depression, autism spectrum, cardiac health and blood pressure, tinnitus, cognitive impairment, and for its neuroprotective effects Ideal for patients with glutathione deficiency
Omega-3 fatty acids	Omega-3 fatty acids may improve cognition in patients with ADHD, as a result of its effects on the cholinergic system Beneficial for emotional self-regulation
SAMe	Tolerability issues associated with SAMe supplementation due to uncontrolled production of monoamines May or may not be used with cofactors, depending on clinician preference
Vitamin B_12_	Serum levels may be measurable (>500 ng/mL) but MMA levels may be high; levels should not exceed 100
Zinc	Low serum levels of zinc correlate with cognitive impairment and anxiety

ADHD, attention-deficit/hyperactivity disorder; BDNF, brain-derived neurotrophic factor; CoQ10, coenzyme Q 10; MMA, methylmalonic acid; *MTHFR*, methylenetetrahydrofolate reductase; NAC, N-acetylcysteine; OCD, obsessive-compulsive disorder; SAMe, *S*-adenosylmethionine; TMG, trimethylglycine.

## Data Availability

Not Applicable.

## Abbreviations

Acyl-CoA	acyl-coenzyme A
AE	adverse event
ALA	alpha-lipoic acid
ATP	adenosine triphosphate
BBB	blood-brain barrier
BMI	body mass index
CGI	Clinical Global Impressions
CGI-S	Clinical Global Impressions-Severity of Illness
CI	confidence interval
CNS	central nervous system
*COMT*	catechol-*O*-methyltransferase
CoQ10	coenzyme Q10
CRP	C-reactive protein
DAG	diacylglycerol
DHLA	dihydrolipoic acid
EPA	eicosapentaenoic acid
HAM-D	Hamilton Depression Rating Scale
HPA	hypothalamic-pituitary-adrenal
HR	hazard ratio
hsCRP	high-sensitivity C-reactive protein
IL-8	interleukin-8
IP3	inositol triphosphate
LAC	L-acetylcarnitine
LCFA	long chain fatty acids
MADRS	Montgomery–Åsberg Depression Rating Scale
MAOIs	monoamine oxidase inhibitors
MAPK	mitogen activated protein kinase
MDD	major depressive disorder
mGlu 2	type 2 metabotropic glutamate
mGlu	metabotropic glutamate
MMA	methylmalonic acid
MTHF	L-5-methyl-tetrahydrofolate
MTHFR	5,10-methylenetetrahydrofolate reductase
NAC	N-acetylcysteine
NMDA	N-methyl-D-aspartate
OR	odds ratio
PIP	phosphatidylinositol
PUFAs	polyunsaturated fatty acids
RR	relative risk
SAH	*S*-adenosylhomocysteine
SAMe	*S*-adenosylmethionine
SD	standard deviation
SMD	standardized mean difference
SNRIs	serotonin norepinephrine reuptake inhibitors
SSRIs	selective serotonin reuptake inhibitors
STAR*D	Sequenced Treatment Alternatives to Relieve Depression
TCAs	tricyclic antidepressants
TDO	tryptophan 2,3-dioxygenase
TNF-α	tumor necrosis factor-α
VDRs	vitamin D receptors

## References

[1] Uher R., Payne J.L., Pavlova B., Perlis R.H. (2014). Major depressive disorder in DSM-5: Implications for clinical practice and research of changes from DSM-IV. Depress. Anxiety.

[2] Morrissette D.A., Stahl S.M. (2014). Modulating the serotonin system in the treatment of major depressive disorder. CNS Spectr..

[3] Blier P., El Mansari M. (2013). Serotonin and beyond: Therapeutics for major depression. Philos. Trans. R. Soc. Lond. B Biol. Sci..

[4] Ceskova E., Silhan P. (2018). Novel treatment options in depression and psychosis. Neuropsychiatr. Dis. Treat..

[5] Benkelfat C., Ellenbogen M.A., Dean P., Palmour R.M., Young S.N. (1994). Mood-lowering effect of tryptophan depletion. Enhanced susceptibility in young men at genetic risk for major affective disorders. Arch. Gen. Psychiatry.

[6] Nordstrom P., Samuelsson M., Asberg M., Traskman-Bendz L., Aberg-Wistedt A., Nordin C., Bertilsson L. (1994). CSF 5-HIAA predicts suicide risk after attempted suicide. Suicide Life-Threat. Behav..

[7] Baldwin D., Rudge S. (1995). The role of serotonin in depression and anxiety. Int. Clin. Psychopharmacol..

[8] Stahl S.M. (2013). Stahl’s Essential Psychopharmacology: Neuroscientific Basis and Practical Application.

[9] Lim S.Y., Kim E.J., Kim A., Lee H.J., Choi H.J., Yang S.J. (2016). Nutritional Factors Affecting Mental Health. Clin. Nutr. Res..

[10] Bottiglieri T., Laundy M., Crellin R., Toone B.K., Carney M.W., Reynolds E.H. (2000). Homocysteine, folate, methylation, and monoamine metabolism in depression. J. Neurol. Neurosurg. Psychiatry.

[11] Godfrey P.S., Toone B.K., Carney M.W., Flynn T.G., Bottiglieri T., Laundy M., Chanarin I., Reynolds E.H. (1990). Enhancement of recovery from psychiatric illness by methylfolate. Lancet.

[12] Jacka F.N., Maes M., Pasco J.A., Williams L.J., Berk M. (2012). Nutrient intakes and the common mental disorders in women. J. Affect. Disord..

[13] Lang U.E., Beglinger C., Schweinfurth N., Walter M., Borgwardt S. (2015). Nutritional aspects of depression. Cell. Physiol. Biochem..

[14] Jeon S.W., Kim Y.K. (2018). The role of neuroinflammation and neurovascular dysfunction in major depressive disorder. J. Inflamm. Res..

[15] Rosenblat J.D., Cha D.S., Mansur R.B., McIntyre R.S. (2014). Inflamed moods: A review of the interactions between inflammation and mood disorders. Prog. Neuro-Psychopharmacol. Biol. Psychiatry.

[16] Hurley L.L., Tizabi Y. (2013). Neuroinflammation, neurodegeneration, and depression. Neurotox. Res..

[17] Raison C.L., Miller A.H. (2013). Role of inflammation in depression: Implications for phenomenology, pathophysiology and treatment. Mod. Trends Pharm..

[18] Miller A.H., Maletic V., Raison C.L. (2009). Inflammation and its discontents: The role of cytokines in the pathophysiology of major depression. Biol. Psychiatry.

[19] Howren M.B., Lamkin D.M., Suls J. (2009). Associations of depression with C-reactive protein, IL-1, and IL-6: A meta-analysis. Psychosom. Med..

[20] Dowlati Y., Herrmann N., Swardfager W., Liu H., Sham L., Reim E.K., Lanctot K.L. (2010). A meta-analysis of cytokines in major depression. Biol. Psychiatry.

[21] Yin L., Xu X., Chen G., Mehta N.D., Haroon E., Miller A.H., Luo Y., Li Z., Felger J.C. (2019). Inflammation and decreased functional connectivity in a widely-distributed network in depression: Centralized effects in the ventral medial prefrontal cortex. Brain Behav. Immun..

[22] Liu Y., Ho R.C., Mak A. (2012). Interleukin (IL)-6, tumour necrosis factor alpha (TNF-alpha) and soluble interleukin-2 receptors (sIL-2R) are elevated in patients with major depressive disorder: A meta-analysis and meta-regression. J. Affect. Disord..

[23] Maletic V., Raison C. (2017). The New Mind-Body Science of Depression.

[24] Mill J., Petronis A. (2007). Molecular studies of major depressive disorder: The epigenetic perspective. Mol. Psychiatry.

[25] Nasca C., Xenos D., Barone Y., Caruso A., Scaccianoce S., Matrisciano F., Battaglia G., Mathe A.A., Pittaluga A., Lionetto L. (2013). L-acetylcarnitine causes rapid antidepressant effects through the epigenetic induction of mGlu2 receptors. Proc. Natl. Acad. Sci. USA.

[26] Nestler E.J. (2014). Epigenetic mechanisms of depression. JAMA Psychiatry.

[27] Pena C.J., Nestler E.J. (2018). Progress in epigenetics of depression. Prog. Mol. Biol. Transl. Sci..

[28] Bottiglieri T. (2013). Folate, vitamin B(1)(2), and S-adenosylmethionine. Psychiatr. Clin. N. Am..

[29] Black M.M. (2008). Effects of vitamin B12 and folate deficiency on brain development in children. Food Nutr. Bull..

[30] Nanri A., Hayabuchi H., Ohta M., Sato M., Mishima N., Mizoue T. (2012). Serum folate and depressive symptoms among Japanese men and women: A cross-sectional and prospective study. Psychiatry Res..

[31] Gilbody S., Lightfoot T., Sheldon T. (2007). Is low folate a risk factor for depression? A meta-analysis and exploration of heterogeneity. J. Epidemiol. Community Health.

[32] Kiecolt-Glaser J.K., Derry H.M., Fagundes C.P. (2015). Inflammation: Depression fans the flames and feasts on the heat. Am. J. Psychiatry.

[33] Kopschina Feltes P., Doorduin J., Klein H.C., Juarez-Orozco L.E., Dierckx R.A., Moriguchi-Jeckel C.M., de Vries E.F. (2017). Anti-inflammatory treatment for major depressive disorder: Implications for patients with an elevated immune profile and non-responders to standard antidepressant therapy. J. Psychopharmacol..

[34] Miller A.H., Haroon E., Raison C.L., Felger J.C. (2013). Cytokine targets in the brain: Impact on neurotransmitters and neurocircuits. Depress. Anxiety.

[35] Alpert J.E., Fava M. (1997). Nutrition and depression: The role of folate. Nutr. Rev..

[36] Stahl S.M. (2008). L-methylfolate: A vitamin for your monoamines. J. Clin. Psychiatry.

[37] Pietrzik K., Bailey L., Shane B. (2010). Folic acid and L-5-methyltetrahydrofolate: Comparison of clinical pharmacokinetics and pharmacodynamics. Clin. Pharmacokinet..

[38] Bottiglieri T. (2005). Homocysteine and folate metabolism in depression. Prog. Neuro-Psychopharmacol. Biol. Psychiatry.

[39] Dean O., Giorlando F., Berk M. (2011). N-acetylcysteine in psychiatry: Current therapeutic evidence and potential mechanisms of action. J. Psychiatry Neurosci..

[40] Fries G.R., Kapczinski F. (2011). N-acetylcysteine as a mitochondrial enhancer: A new class of psychoactive drugs?. Braz. J. Psychiatry.

[41] Budni J., Zomkowski A.D., Engel D., Santos D.B., dos Santos A.A., Moretti M., Valvassori S.S., Ornell F., Quevedo J., Farina M. (2013). Folic acid prevents depressive-like behavior and hippocampal antioxidant imbalance induced by restraint stress in mice. Exp. Neurol..

[42] Papakostas G.I., Shelton R.C., Zajecka J.M., Etemad B., Rickels K., Clain A., Baer L., Dalton E.D., Sacco G.R., Schoenfeld D. (2012). L-methylfolate as adjunctive therapy for SSRI-resistant major depression: Results of two randomized, double-blind, parallel-sequential trials. Am. J. Psychiatry.

[43] (2017). Deplin Capsules [Package Insert].

[44] Papakostas G.I., Shelton R.C., Zajecka J.M., Bottiglieri T., Roffman J., Cassiello C., Stahl S.M., Fava M. (2014). Effect of adjunctive L-methylfolate 15 mg among inadequate responders to SSRIs in depressed patients who were stratified by biomarker levels and genotype: Results from a randomized clinical trial. J. Clin. Psychiatry.

[45] Shelton R.C., Pencina M.J., Barrentine L.W., Ruiz J.A., Fava M., Zajecka J.M., Papakostas G.I. (2015). Association of obesity and inflammatory marker levels on treatment outcome: Results from a double-blind, randomized study of adjunctive L-methylfolate calcium in patients with MDD who are inadequate responders to SSRIs. J. Clin. Psychiatry.

[46] Ginsberg L.D., Oubre A.Y., Daoud Y.A. (2011). L-methylfolate Plus SSRI or SNRI from Treatment Initiation Compared to SSRI or SNRI Monotherapy in a Major Depressive Episode. Innov. Clin. Neurosci..

[47] Papakostas G.I., Mischoulon D., Shyu I., Alpert J.E., Fava M. (2010). S-adenosyl methionine (SAMe) augmentation of serotonin reuptake inhibitors for antidepressant nonresponders with major depressive disorder: A double-blind, randomized clinical trial. Am. J. Psychiatry.

[48] Williams A.L., Girard C., Jui D., Sabina A., Katz D.L. (2005). S-adenosylmethionine (SAMe) as treatment for depression: A systematic review. Clin. Investig. Med. Med. Clin. Exp..

[49] Galizia I., Oldani L., Macritchie K., Amari E., Dougall D., Jones T.N., Lam R.W., Massei G.J., Yatham L.N., Young A.H. (2016). S-adenosyl methionine (SAMe) for depression in adults. Cochrane Database Syst. Rev..

[50] Nasca C., Bigio B., Lee F.S., Young S.P., Kautz M.M., Albright A., Beasley J., Millington D.S., Mathe A.A., Kocsis J.H. (2018). Acetyl-l-carnitine deficiency in patients with major depressive disorder. Proc. Natl. Acad. Sci. USA.

[51] Veronese N., Stubbs B., Solmi M., Ajnakina O., Carvalho A.F., Maggi S. (2018). Acetyl-L-carnitine supplementation and the treatment of depressive symptoms: A systematic review and meta-analysis. Psychosom. Med..

[52] Brennan B.P., Jensen J.E., Hudson J.I., Coit C.E., Beaulieu A., Pope H.G., Renshaw P.F., Cohen B.M. (2013). A placebo-controlled trial of acetyl-L-carnitine and alpha-lipoic acid in the treatment of bipolar depression. J. Clin. Psychopharmacol..

[53] Zheng W., Zhang Q.E., Cai D.B., Yang X.H., Qiu Y., Ungvari G.S., Ng C.H., Berk M., Ning Y.P., Xiang Y.T. (2018). N-acetylcysteine for major mental disorders: A systematic review and meta-analysis of randomized controlled trials. Acta Psychiatr. Scand..

[54] Fernandes B.S., Dean O.M., Dodd S., Malhi G.S., Berk M. (2016). N-Acetylcysteine in depressive symptoms and functionality: A systematic review and meta-analysis. J. Clin. Psychiatry.

[55] Berk M., Dean O.M., Cotton S.M., Jeavons S., Tanious M., Kohlmann K., Hewitt K., Moss K., Allwang C., Schapkaitz I. (2014). The efficacy of adjunctive N-acetylcysteine in major depressive disorder: A double-blind, randomized, placebo-controlled trial. J. Clin. Psychiatry.

[56] Magalhaes P.V., Dean O.M., Bush A.I., Copolov D.L., Malhi G.S., Kohlmann K., Jeavons S., Schapkaitz I., Anderson-Hunt M., Berk M. (2011). N-acetylcysteine for major depressive episodes in bipolar disorder. Braz. J. Psychiatry.

[57] Siwek M., Dudek D., Schlegel-Zawadzka M., Morawska A., Piekoszewski W., Opoka W., Zieba A., Pilc A., Popik P., Nowak G. (2010). Serum zinc level in depressed patients during zinc supplementation of imipramine treatment. J. Affect. Disord..

[58] Maserejian N.N., Hall S.A., McKinlay J.B. (2012). Low dietary or supplemental zinc is associated with depression symptoms among women, but not men, in a population-based epidemiological survey. J. Affect. Disord..

[59] Swardfager W., Herrmann N., Mazereeuw G., Goldberger K., Harimoto T., Lanctot K.L. (2013). Zinc in depression: A meta-analysis. Biol. Psychiatry.

[60] Tarleton E.K., Littenberg B., MacLean C.D., Kennedy A.G., Daley C. (2017). Role of magnesium supplementation in the treatment of depression: A randomized clinical trial. PLoS ONE.

[61] Spedding S. (2014). Vitamin D and depression: A systematic review and meta-analysis comparing studies with and without biological flaws. Nutrients.

[62] Kelly C.B., McDonnell A.P., Johnston T.G., Mulholland C., Cooper S.J., McMaster D., Evans A., Whitehead A.S. (2004). The MTHFR C677T polymorphism is associated with depressive episodes in patients from Northern Ireland. J. Psychopharmacol..

[63] Lok A., Bockting C.L., Koeter M.W., Snieder H., Assies J., Mocking R.J., Vinkers C.H., Kahn R.S., Boks M.P., Schene A.H. (2013). Interaction between the MTHFR C677T polymorphism and traumatic childhood events predicts depression. Transl. Psychiatry.

[64] Wu Y.L., Ding X.X., Sun Y.H., Yang H.Y., Chen J., Zhao X., Jiang Y.H., Lv X.L., Wu Z.Q. (2013). Association between MTHFR C677T polymorphism and depression: An updated meta-analysis of 26 studies. Prog. Neuro-Psychopharmacol. Biol. Psychiatry.

[65] Shelton R.C., Sloan Manning J., Barrentine L.W., Tipa E.V. (2013). Assessing Effects of l-Methylfolate in Depression Management: Results of a Real-World Patient Experience Trial. Prim. Care Companion CNS Disord..

[66] Dartois L.L., Stutzman D.L., Morrow M. (2019). L-methylfolate Augmentation to Antidepressants for Adolescents with Treatment-Resistant Depression: A Case Series. J. Child Adolesc. Psychopharmacol..

[67] Rainka M., Aladeen T., Westphal E., Meaney J., Gengo F., Greger J., Capote H. (2019). L-methylfolate calcium supplementation in adolescents and children: A retrospective analysis. J. Psychiatry Pract..

[68] Zeier Z., Carpenter L.L., Kalin N.H., Rodriguez C.I., McDonald W.M., Widge A.S., Nemeroff C.B. (2018). Clinical implementation of pharmacogenetic decision support tools for antidepressant drug prescribing. Am. J. Psychiatry.

[69] Poddar R., Sivasubramanian N., DiBello P.M., Robinson K., Jacobsen D.W. (2001). Homocysteine induces expression and secretion of monocyte chemoattractant protein-1 and interleukin-8 in human aortic endothelial cells: Implications for vascular disease. Circulation.

[70] Folstein M., Liu T., Peter I., Buell J., Arsenault L., Scott T., Qiu W.W. (2007). The homocysteine hypothesis of depression. Am. J. Psychiatry.

[71] Clarke R., Smith A.D., Jobst K.A., Refsum H., Sutton L., Ueland P.M. (1998). Folate, vitamin B12, and serum total homocysteine levels in confirmed Alzheimer disease. Arch. Neurol..

[72] Hasan T., Arora R., Bansal A.K., Bhattacharya R., Sharma G.S., Singh L.R. (2019). Disturbed homocysteine metabolism is associated with cancer. Exp. Mol. Med..

[73] Vacek T.P., Kalani A., Voor M.J., Tyagi S.C., Tyagi N. (2013). The role of homocysteine in bone remodeling. Clin. Chem. Lab. Med..

[74] Wedro B. Homocysteine (Blood Test). https://www.emedicinehealth.com/homocysteine/article_em.htm.

[75] Beard R.S., Reynolds J.J., Bearden S.E. (2011). Hyperhomocysteinemia increases permeability of the blood-brain barrier by NMDA receptor-dependent regulation of adherens and tight junctions. Blood.

[76] Chung K.H., Chiou H.Y., Chen Y.H. (2017). Associations between serum homocysteine levels and anxiety and depression among children and adolescents in Taiwan. Sci. Rep..

[77] Bjelland I., Tell G.S., Vollset S.E., Refsum H., Ueland P.M. (2003). Folate, vitamin B12, homocysteine, and the MTHFR 677C->T polymorphism in anxiety and depression: The Hordaland Homocysteine Study. Arch. Gen. Psychiatry.

[78] Gariballa S. (2011). Testing homocysteine-induced neurotransmitter deficiency, and depression of mood hypothesis in clinical practice. Age Ageing.

[79] Permoda-Osip A., Kisielewski J., Dorszewska J., Rybakowski J. (2014). Homocysteine and cognitive functions in bipolar depression. Psychiatr. Pol..

[80] Klee G.G. (2000). Cobalamin and folate evaluation: Measurement of methylmalonic acid and homocysteine vs. vitamin B(12) and folate. Clin. Chem..

[81] Sharma A., Gerbarg P., Bottiglieri T., Massoumi L., Carpenter L.L., Lavretsky H., Muskin P.R., Brown R.P., Mischoulon D. (2017). S-Adenosylmethionine (SAMe) for Neuropsychiatric Disorders: A Clinician-Oriented Review of Research. J. Clin. Psychiatry.

[82] Gelenberg A.J., Freeman M.P., Markowitz J.C., Rosenbaum J.F., Thase M.E., Trivedi M.H., Van Rhoads R.S. Practice Guideline for the Treatment of Patients with Major Depressive Disorder. http://psychiatryonline.org/pb/assets/raw/sitewide/practice_guidelines/guidelines/mdd.pdf.

[83] Mischoulon D., Fava M. (2002). Role of S-adenosyl-L-methionine in the treatment of depression: A review of the evidence. Am. J. Clin. Nutr..

[84] Papakostas G.I., Cassiello C.F., Iovieno N. (2012). Folates and S-adenosylmethionine for major depressive disorder. Can. J. Psychiatry.

[85] Menke A., Binder E.B. (2014). Epigenetic alterations in depression and antidepressant treatment. Dialogues Clin. Neurosci..

[86] Ferreira G.C., McKenna M.C. (2017). L-Carnitine and Acetyl-L-carnitine Roles and Neuroprotection in Developing Brain. Neurochem Res.

[87] Soczynska J.K., Kennedy S.H., Chow C.S., Woldeyohannes H.O., Konarski J.Z., McIntyre R.S. (2008). Acetyl-L-carnitine and alpha-lipoic acid: Possible neurotherapeutic agents for mood disorders?. Expert Opin. Investig. Drugs.

[88] Wang W., Lu Y., Xue Z., Li C., Wang C., Zhao X., Zhang J., Wei X., Chen X., Cui W. (2015). Rapid-acting antidepressant-like effects of acetyl-l-carnitine mediated by PI3K/AKT/BDNF/VGF signaling pathway in mice. Neuroscience.

[89] Shay K.P., Moreau R.F., Smith E.J., Smith A.R., Hagen T.M. (2009). Alpha-lipoic acid as a dietary supplement: Molecular mechanisms and therapeutic potential. Biochim. Biophys. Acta.

[90] Gilgun-Sherki Y., Melamed E., Offen D. (2001). Oxidative stress induced-neurodegenerative diseases: The need for antioxidants that penetrate the blood brain barrier. Neuropharmacology.

[91] Packer L., Witt E.H., Tritschler H.J. (1995). Alpha-Lipoic acid as a biological antioxidant. Free Radic. Biol. Med..

[92] Parcell S. (2002). Sulfur in human nutrition and applications in medicine. Altern. Med. Rev..

[93] Zhao G., Etherton T.D., Martin K.R., Gillies P.J., West S.G., Kris-Etherton P.M. (2007). Dietary alpha-linolenic acid inhibits proinflammatory cytokine production by peripheral blood mononuclear cells in hypercholesterolemic subjects. Am. J. Clin. Nutr..

[94] Zhang Y., Han P., Wu N., He B., Lu Y., Li S., Liu Y., Zhao S., Liu L., Li Y. (2011). Amelioration of lipid abnormalities by alpha-lipoic acid through antioxidative and anti-inflammatory effects. Obesity.

[95] Sola S., Mir M.Q., Cheema F.A., Khan-Merchant N., Menon R.G., Parthasarathy S., Khan B.V. (2005). Irbesartan and lipoic acid improve endothelial function and reduce markers of inflammation in the metabolic syndrome: Results of the Irbesartan and Lipoic Acid in Endothelial Dysfunction (ISLAND) study. Circulation.

[96] Silva M.C., de Sousa C.N., Sampaio L.R., Ximenes N.C., Araujo P.V., da Silva J.C., de Oliveira S.L., Sousa F.C., Macedo D.S., Vasconcelos S.M. (2013). Augmentation therapy with alpha-lipoic acid and desvenlafaxine: A future target for treatment of depression?. Naunyn Schmiedebergs Arch. Pharmacol..

[97] Aliev G., Liu J., Shenk J.C., Fischbach K., Pacheco G.J., Chen S.G., Obrenovich M.E., Ward W.F., Richardson A.G., Smith M.A. (2009). Neuronal mitochondrial amelioration by feeding acetyl-L-carnitine and lipoic acid to aged rats. J. Cell. Mol. Med..

[98] Liu J., Head E., Gharib A.M., Yuan W., Ingersoll R.T., Hagen T.M., Cotman C.W., Ames B.N. (2002). Memory loss in old rats is associated with brain mitochondrial decay and RNA/DNA oxidation: Partial reversal by feeding acetyl-L-carnitine and/or R-alpha -lipoic acid. Proc. Natl. Acad. Sci. USA.

[99] Hagen T.M., Liu J., Lykkesfeldt J., Wehr C.M., Ingersoll R.T., Vinarsky V., Bartholomew J.C., Ames B.N. (2002). Feeding acetyl-L-carnitine and lipoic acid to old rats significantly improves metabolic function while decreasing oxidative stress. Proc. Natl. Acad. Sci. USA.

[100] Noto C., Rizzo L.B., Mansur R.B., McIntyre R.S., Maes M., Brietzke E. (2014). Targeting the inflammatory pathway as a therapeutic tool for major depression. Neuroimmunomodulation.

[101] Deepmala, Slattery J., Kumar N., Delhey L., Berk M., Dean O., Spielholz C., Frye R. (2015). Clinical trials of N-acetylcysteine in psychiatry and neurology: A systematic review. Neurosci. Biobehav. Rev..

[102] Erickson M.A., Hansen K., Banks W.A. (2012). Inflammation-induced dysfunction of the low-density lipoprotein receptor-related protein-1 at the blood-brain barrier: Protection by the antioxidant N-acetylcysteine. Brain Behav. Immun..

[103] Ooi S.L., Green R., Pak S.C. (2018). N-Acetylcysteine for the Treatment of Psychiatric Disorders: A Review of Current Evidence. Biomed Res. Int..

[104] Wigner P., Czarny P., Galecki P., Sliwinski T. (2017). Oxidative and Nitrosative Stress as Well as the Tryptophan Catabolites Pathway in Depressive Disorders. Psychiatr. Danub..

[105] Myint A.M., Kim Y.K., Verkerk R., Scharpe S., Steinbusch H., Leonard B. (2007). Kynurenine pathway in major depression: Evidence of impaired neuroprotection. J. Affect. Disord..

[106] Young S.N., Leyton M. (2002). The role of serotonin in human mood and social interaction. Insight from altered tryptophan levels. Pharmacol. Biochem. Behav..

[107] Moreno F.A., Gelenberg A.J., Heninger G.R., Potter R.L., McKnight K.M., Allen J., Phillips A.P., Delgado P.L. (1999). Tryptophan depletion and depressive vulnerability. Biol. Psychiatry.

[108] Leyton M., Ghadirian A.M., Young S.N., Palmour R.M., Blier P., Helmers K.F., Benkelfat C. (2000). Depressive relapse following acute tryptophan depletion in patients with major depressive disorder. J. Psychopharmacol..

[109] Maes M., Meltzer H.Y., Scharpe S., Bosmans E., Suy E., De Meester I., Calabrese J., Cosyns P. (1993). Relationships between lower plasma L-tryptophan levels and immune-inflammatory variables in depression. Psychiatry Res..

[110] Anderson G., Kubera M., Duda W., Lason W., Berk M., Maes M. (2013). Increased IL-6 trans-signaling in depression: Focus on the tryptophan catabolite pathway, melatonin and neuroprogression. Pharmacol. Rep..

[111] Fernstrom J.D. (2012). Effects and side effects associated with the non-nutritional use of tryptophan by humans. J. Nutr..

[112] Lindseth G., Helland B., Caspers J. (2015). The effects of dietary tryptophan on affective disorders. Arch. Psychiatry Nurs..

[113] Foong A.L., Patel T., Kellar J., Grindrod K.A. (2018). The scoop on serotonin syndrome. Can. Pharm. J..

[114] Wang J., Um P., Dickerman B.A., Liu J. (2018). Zinc, Magnesium, Selenium and Depression: A Review of the Evidence, Potential Mechanisms and Implications. Nutrients.

[115] Styczen K., Sowa-Kucma M., Siwek M., Dudek D., Reczynski W., Szewczyk B., Misztak P., Topor-Madry R., Opoka W., Nowak G. (2017). The serum zinc concentration as a potential biological marker in patients with major depressive disorder. Metab. Brain Dis..

[116] Mlyniec K. (2015). Zinc in the Glutamatergic Theory of Depression. Curr. Neuropharmacol..

[117] Swardfager W., Herrmann N., McIntyre R.S., Mazereeuw G., Goldberger K., Cha D.S., Schwartz Y., Lanctot K.L. (2013). Potential roles of zinc in the pathophysiology and treatment of major depressive disorder. Neurosci. Biobehav. Rev..

[118] Takeda A., Tamano H., Nishio R., Murakami T. (2016). Behavioral Abnormality Induced by Enhanced Hypothalamo-Pituitary-Adrenocortical Axis Activity under Dietary Zinc Deficiency and Its Usefulness as a Model. Int. J. Mol. Sci..

[119] Serefko A., Szopa A., Wlaz P., Nowak G., Radziwon-Zaleska M., Skalski M., Poleszak E. (2013). Magnesium in depression. Pharmacol. Rep..

[120] Hightower J.M., Dalessandri K.M., Pope K., Hernandez G.T. (2017). Low 25-Hydroxyvitamin D and Myofascial Pain: Association of Cancer, Colon Polyps, and Tendon Rupture. J. Am. Coll. Nutr..

[121] McDonald J.W., Silverstein F.S., Johnston M.V. (1990). Magnesium reduces N-methyl-D-aspartate (NMDA)-mediated brain injury in perinatal rats. Neurosci. Lett..

[122] Zarate C.A., Mathews D.C., Furey M.L. (2013). Human biomarkers of rapid antidepressant effects. Biol. Psychiatry.

[123] Tarleton E.K., Littenberg B. (2015). Magnesium intake and depression in adults. J. Am. Board Fam. Med..

[124] Eby G.A., Eby K.L. (2006). Rapid recovery from major depression using magnesium treatment. Med. Hypotheses.

[125] Poleszak E., Szewczyk B., Kedzierska E., Wlaz P., Pilc A., Nowak G. (2004). Antidepressant- and anxiolytic-like activity of magnesium in mice. Pharmacol. Biochem. Behav..

[126] Poleszak E., Wlaz P., Kedzierska E., Radziwon-Zaleska M., Pilc A., Fidecka S., Nowak G. (2005). Effects of acute and chronic treatment with magnesium in the forced swim test in rats. Pharmacol. Rep..

[127] Ganji V., Milone C., Cody M.M., McCarty F., Wang Y.T. (2010). Serum vitamin D concentrations are related to depression in young adult US population: The Third National Health and Nutrition Examination Survey. Int. Arch. Med..

[128] Cherniack E.P., Troen B.R., Florez H.J., Roos B.A., Levis S. (2009). Some new food for thought: The role of vitamin D in the mental health of older adults. Curr. Psychiatry Rep..

[129] Anglin R.E., Samaan Z., Walter S.D., McDonald S.D. (2013). Vitamin D deficiency and depression in adults: Systematic review and meta-analysis. Br. J. Psychiatry.

[130] Bertone-Johnson E.R. (2009). Vitamin D and the occurrence of depression: Causal association or circumstantial evidence?. Nutr. Rev..

[131] Wilkins C.H., Sheline Y.I., Roe C.M., Birge S.J., Morris J.C. (2006). Vitamin D deficiency is associated with low mood and worse cognitive performance in older adults. Am. J. Geriatr. Psychiatry.

[132] Milaneschi Y., Hoogendijk W., Lips P., Heijboer A.C., Schoevers R., van Hemert A.M., Beekman A.T., Smit J.H., Penninx B.W. (2014). The association between low vitamin D and depressive disorders. Mol. Psychiatry.

[133] Holick M.F. (2007). Vitamin D deficiency. N. Engl. J. Med..

[134] Bischoff-Ferrari H.A., Shao A., Dawson-Hughes B., Hathcock J., Giovannucci E., Willett W.C. (2010). Benefit-risk assessment of vitamin D supplementation. Osteoporos. Int..

[135] Vieth R. (2007). Vitamin D toxicity, policy, and science. J. Bone Miner. Res..

[136] Peet M., Stokes C. (2005). Omega-3 fatty acids in the treatment of psychiatric disorders. Drugs.

[137] Wani A.L., Bhat S.A., Ara A. (2015). Omega-3 fatty acids and the treatment of depression: A review of scientific evidence. Integr. Med. Res..

[138] Sinclair A.J., Begg D., Mathai M., Weisinger R.S. (2007). Omega 3 fatty acids and the brain: Review of studies in depression. Asia Pac J. Clin. Nutr..

[139] Grosso G., Galvano F., Marventano S., Malaguarnera M., Bucolo C., Drago F., Caraci F. (2014). Omega-3 fatty acids and depression: Scientific evidence and biological mechanisms. Oxidative Med. Cell. Longev..

[140] Labrousse V.F., Nadjar A., Joffre C., Costes L., Aubert A., Gregoire S., Bretillon L., Laye S. (2012). Short-term long chain omega3 diet protects from neuroinflammatory processes and memory impairment in aged mice. PLoS ONE.

[141] Larrieu T., Laye S. (2018). Food for Mood: Relevance of Nutritional Omega-3 Fatty Acids for Depression and Anxiety. Front. Physiol..

[142] McNamara R.K., Jandacek R., Rider T., Tso P., Cole-Strauss A., Lipton J.W. (2010). Omega-3 fatty acid deficiency increases constitutive pro-inflammatory cytokine production in rats: Relationship with central serotonin turnover. Prostaglandins Leukot. Essent. Fat. Acids.

[143] Feart C., Peuchant E., Letenneur L., Samieri C., Montagnier D., Fourrier-Reglat A., Barberger-Gateau P. (2008). Plasma eicosapentaenoic acid is inversely associated with severity of depressive symptomatology in the elderly: Data from the Bordeaux sample of the Three-City Study. Am. J. Clin. Nutr..

[144] Edwards R., Peet M., Shay J., Horrobin D. (1998). Omega-3 polyunsaturated fatty acid levels in the diet and in red blood cell membranes of depressed patients. J. Affect. Disord..

[145] Rapaport M.H., Nierenberg A.A., Schettler P.J., Kinkead B., Cardoos A., Walker R., Mischoulon D. (2016). Inflammation as a predictive biomarker for response to omega-3 fatty acids in major depressive disorder: A proof-of-concept study. Mol. Psychiatry.

[146] Deane K.H.O., Jimoh O.F., Biswas P., O’Brien A., Hanson S., Abdelhamid A.S., Fox C., Hooper L. (2019). Omega-3 and polyunsaturated fat for prevention of depression and anxiety symptoms: Systematic review and meta-analysis of randomised trials. Br. J. Psychiatry.

[147] Maes M., Mihaylova I., Kubera M., Uytterhoeven M., Vrydags N., Bosmans E. (2009). Lower plasma Coenzyme Q10 in depression: A marker for treatment resistance and chronic fatigue in depression and a risk factor to cardiovascular disorder in that illness. Neuro. Endocrinol. Lett..

[148] Morris G., Anderson G., Berk M., Maes M. (2013). Coenzyme Q10 depletion in medical and neuropsychiatric disorders: Potential repercussions and therapeutic implications. Mol. Neurobiol..

[149] Forester B.P., Harper D.G., Georgakas J., Ravichandran C., Madurai N., Cohen B.M. (2015). Antidepressant effects of open label treatment with coenzyme Q10 in geriatric bipolar depression. J. Clin. Psychopharmacol..

